# IDEAS for a healthy baby - reducing disparities in use of publicly reported quality data: study protocol for a randomized controlled trial

**DOI:** 10.1186/1745-6215-14-244

**Published:** 2013-08-07

**Authors:** Sarah L Goff, Penelope S Pekow, Katharine O White, Tara Lagu, Kathleen M Mazor, Peter K Lindenauer

**Affiliations:** 1 Center for Quality of Care Research, Baystate Medical Center, 280 Chestnut St., Room 305, Springfield, MA 01199, USA; 2 Department of Medicine, Baystate Medical Center Springfield, MA and Tufts University School of Medicine, Boston, MA, USA; 3 Department of Obstetrics and Gynecology, Baystate Medical Center and Tufts University School of Medicine, Boston, MA, USA; 4 Department of Epidemiology and Biostatistics, University of Massachusetts, Amherst, MA, USA; 5 University of Massachusetts Medical Center, and Meyers Primary Care Institute, Worcester, MA, USA

**Keywords:** Publicly reported quality data, Pediatric, Patient navigator, Pregnancy, Intervention studies, Randomized trials

## Abstract

**Background:**

Publicly reported performance on quality measures is intended to enable patients to make more informed choices. Despite the growing availability of these reports, patients’ use remains limited and disparities exist. Low health literacy and numeracy are two barriers that may contribute to these disparities. Patient navigators have helped patients overcome barriers such as these in other areas, such as cancer care and may prove useful for overcoming barriers to using publicly reported quality data.

**Methods/Design:**

The goals of this study are: to determine the efficacy of a patient navigator intervention to assist low-income pregnant women in the use of publicly available information about quality of care when choosing a pediatrician; to evaluate the relative importance of factors influencing women’s choice of pediatric practices; to evaluate the effect of the intervention on patient engagement in management of their own and their child’s health care; and to assess variation in efficacy of the intervention for sub-groups based on parity, age, and race/ethnicity. English speaking women ages 16 to 50 attending a prenatal clinic at a large urban medical center will be randomized to receive an in-person navigator intervention or an informational pamphlet control between 20 to 34 weeks of gestation. The intervention will include in-person guided use of the Massachusetts Health Quality Partners website, which reports pediatric practices’ performance on quality measures and patient experience. The primary study outcomes will be the mean scores on a) clinical quality and b) patient experience measures.

**Discussion:**

Successful completion of the study aims will yield important new knowledge about the value of guided website navigation as a strategy to increase the impact of publicly reported quality data and to reduce disparities in use of these data.

**Trial registration:**

ClinicalTrials.gov #NCT01784575

## Background

Every year, more than 4 million children are born in the US [1]. Shortly after delivery, parents face the important task of selecting a pediatrician for their new baby. Receiving recommended care from a pediatric provider can impact child health, and is particularly important in the first two years of life [2-8]. Variation in care quality exists between health plans, communities, hospitals, outpatient practices, and providers [9-11], but patients may not be aware that this variation exists [12].

One way to help patients become aware of differences in physician performance is through the public reporting of quality measures. If patients use information about performance to choose practices with higher quality care or if public reporting stimulates providers to engage in quality improvement activities, public reporting of quality information should result in better patient outcomes [13]. However, the impact on patients’ choices has been limited to date [14], perhaps in part because of a lack of awareness of publicly reported quality data [15]. Even when patients are aware of websites reporting health care quality data, the information may not effectively reach patients because the websites are difficult to navigate and the data are difficult to interpret [16-19]. This failure to reach patients may be especially pronounced in vulnerable populations, such as those with lower health literacy [18,20].

Massachusetts Health Quality Partners (MHQP) is a non-profit organization that has been working to improve care quality in Massachusetts since 1995. The organization works with stakeholders, including patients, physicians, insurance companies, and academic partners to collect and report quality-of-care data in the Commonwealth. Since 2005, MHQP has included a quality reporting tool known as “Quality Insights” on its website [21]. These reports show adult and pediatric practices’ performance on clinical quality and patient experience measures (Table 1). These reports have been recognized for excellence in methodology and accessibility, and are, to our knowledge, the only source of publicly reported, ambulatory pediatric practice quality data in Massachusetts. The website is currently only available in English.

**Table 1:** Categories of pediatric clinical quality and patient experience reported on the MHQP website


**Clinical quality**	*Asthma care*	*Well child visits*	*Pediatric medications and testing*	*Women’s health*	
	Medication for children ages 5- to 11 years	Well visits for children 0 to 15 months of age	Correct testing for strep	Chlamydia screening (ages 16 to 20 years)	
		Well visits for children ages 3 to 6 years	Correct antibiotic use for upper respiratory infections		
		Well visits for adolescents ages 12 to 21 years	Follow-up for children starting medication for ADHD		
**Patient experience**	How well doctors communicate with patients	How well doctors coordinate care	How well doctors know their patients	How well doctors give preventive care advice	Willingness to recommend

*ADHD* Attention deficit hyperactivity disorder.

Patient navigators assist patients in overcoming barriers to achieving care goals. Traditional patient navigators have successfully helped patients navigate complex care environments and achieve better health outcomes [22-24], with those at risk for low health literacy and numeracy benefiting the most [25-27]. These traditional navigator functions may assist patients attempting to access and interpret publicly reported information about quality of care, tailoring the assistance to the patient’s needs. Pregnant women are generally healthy and have ample time to explore options for pediatric care along with multiple contacts with the healthcare system, making this condition ideal for a navigator intervention to support use of publicly reported quality data.

We plan to implement a randomized controlled trial (RCT) design to test the efficacy of a patient navigator intervention. The goal of the intervention is to educate women about the availability and meaning of quality measures in pediatrics and to review information about the quality of local pediatric practices, enabling them to make a more informed decision about where to obtain care for their child.

## Methods/Design

### Study objectives

The primary study outcomes are a) the mean clinical quality score and b) the mean patient experience score of the pediatric practices selected. The study goals are: to determine the efficacy of a patient navigator intervention to assist low-income pregnant women in the use of publicly available information about quality of care when choosing a pediatrician; to evaluate the relative importance of factors influencing women’s choice of pediatric practices; to evaluate the effect of the intervention on patient engagement in management of their own and their child’s health care; and to assess variation in efficacy of the intervention for sub-groups based on parity, age, and race/ethnicity. This study is approved by the Baystate Medical Center Institutional Review Board.

### Study design

This study is a two-arm RCT of patient navigators. Participants are randomized to either the intervention arm (navigator plus informational pamphlet) or the control arm (informational pamphlet only).

### Study site and participants

The Obstetric and Gynecology Clinics at Baystate Medical Center, located in Springfield, Massachusetts, perform approximately 1,500 to 1,800 deliveries annually. The clinics are staffed by eleven obstetricians, six nurse practitioners, and twenty-one house staff from the Baystate Medical Center (BMC)/Tufts University School of Medicine (TUSM) residency training program in obstetrics and gynecology. The majority (89%) of the women attending the clinic are insured through Medicaid, the national public insurance available to women with no employer-based insurance and/or a low-income level [28]. Children of women who qualify for Medicaid often also qualify for this public insurance. A survey undertaken by the American Academy of Pediatric in the year 2000 showed nearly 95% of pediatric practices in Massachusetts accepted all patients with public health insurance [29], but practices may elect to limit the number of patients they take with any type of insurance. Of these, 57% are Hispanic (predominantly from Puerto Rico), 17% are African-American, 23% are non-Hispanic white, and 3% are of other race/ethnicity [28]. Among Hispanic women attending the clinic, nearly 80% prefer speaking English [28]. English-speaking women ages 16 to 50 years between 20 and 34 weeks of pregnancy will be eligible for enrollment in the study.

### Recruitment and informed consent

**Figure 1:**
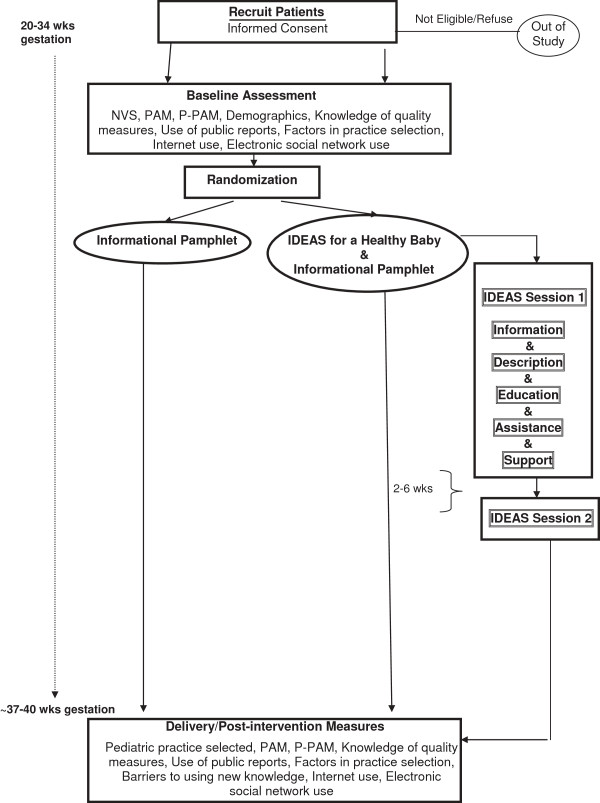
IDEAS (Inform, Describe, Educate, Assist, support) for a healthy baby sessions flow diagram PAM, Patient Activation Measure; P-PAM, Parental Patient Activation Measure; NVS: Newest Vital Sign.

Women will be given a one-page study fact sheet by office staff when they first present to the Wesson Women’s Clinic. When women check in for their prenatal visit during weeks 20 to 34 of gestation, they will be reminded of the study by one of the two trained patient navigators and will be given the one-page fact sheet again as a reminder of study goals. If a woman expresses interest in enrolling, informed consent will then be obtained. Baseline data will be collected and the patient will be randomly assigned to the navigator intervention or the pamphlet control. Randomization will be stratified based on parity because women who have already selected a pediatric practice for their older children may be less likely to change practices based on quality performance alone. If randomized to the intervention arm, the first intervention session will ensue. If in the control arm, the patient will be given an informational pamphlet (described below). If the study process is interrupted, it will be continued at subsequent prenatal visits or by phone when feasible. A flow diagram for the study is shown in Figure 1.

### IDEAS intervention

**Figure 2:**
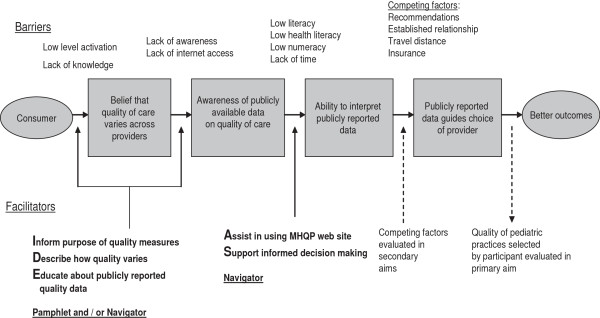
IDEAS for a healthy baby sessions conceptual model.

Patients randomized to the patient navigator intervention will participate in the IDEAS for a Healthy Baby’ sessions. The conceptual model for the intervention is shown in Figure 2. The intervention will include:

•Information about the purpose of quality measures.

•Description of how quality of care varies among practices.

•Education about the existence of publicly reported quality data.

•Assistance in navigating and understanding quality-of-care data on the MHQP website.

•Support for informed decision making in health care.

A list of eligible patients is generated each morning. Patients are then approached by one of the patient navigators in the waiting room of the prenatal clinic and offered an opportunity to participate in the study. If interested, informed consent is obtained and baseline data collected. This may take place in the waiting room, an examination room, or an educational office, when available. Patients randomized to the intervention arm then participate in a brief in-person educational session led by the patient navigator, whose experience includes teaching small undergraduate courses. The navigator’s training for the study included developing an understanding of the methods used to develop the quality measures, use of the MHQP website and obtaining informed consent. Both the primary and secondary navigators participated in extensive rehearsal of the intervention with the principal investigator (PI) feedback prior to piloting then enrolling patients. The information sessions include the rationale for quality measurement, how quality varies among practices, and that information about variation is publicly available. The information session is followed by in-person guided use of the MHQP website with the navigator. Each of two guided website sessions last approximately 20 minutes and take place at two prenatal visits. The first session takes place between 20 and 34 weeks gestation and the second 2 to 6 weeks after the first session. The navigator guides the participant in use of the MHQP website on a laptop computer. There is a standard explanation of the meaning of the quality performance measures and patientexperience survey data. The patient navigator provides the same demonstration to all participants about how the website can be used to assess a practice’s performance. Following this standard intervention, patients are shown performance data for practices they would like to view within a 25-mile radius of their home. If the participant does not have any practices she wishes to view, a standard set of three area practices with high and low scores are shown as examples. Practices shown are recorded. A copy of the study information pamphlet is given after the first guided website session and the participant is asked if they have questions about the pamphlet. During the second intervention session, the patient navigator asks if the participant had an opportunity to look at the website themselves. The navigator then invites them to look at local pediatric practice data again and will respond to any questions about performance data for these practices. Participants may complete a worksheet during the intervention in which they fill out a table of scores on performance measures for the practices they review. Patient navigators do not recommend one practice over another but do discuss the practices’ quality scores and what the scores mean. Objectives of the IDEAS for a healthy baby intervention are outlined in Table 2, along with methods to achieve objectives.

**Table 2:** Objectives and associated navigator actions

**Intervention objective**	**Navigator action**
Inform about the purpose of quality measures	Describes concept of quality in language that is meaningful to participants
Describe how quality of care varies	Gives examples of quality measures and range of performance variation; explains potential meaning for child’s health
Educate about the existence of publicly reported health care quality data	Demonstrates several sites with publicly reported quality data, draws analogy to consumer reports
Assist in use of MHQP website	Shows performance measures on website, describes how they are determined, shows local practice data, guides in use
Support for informed decision making	Puts health care quality in the context of other decisions new parents make (such as circumcisionand breast or bottle feeding)

*MHQP* Massachusetts Health Quality Partners.

### Information pamphlet control

The pamphlet will provide information about clinical quality performance measures, patient satisfaction measures, and information about the MHQP website, including the purpose of the site as well as the web address (URL) where the quality-of-care data relevant to pediatric care are located. Participants randomized to the control group will receive the information pamphlet following collection of baseline measures on the day of enrollment.

### Outcome assessment

All enrolled patients will be interviewed after delivery, prior to hospital discharge for outcome assessment. This assessment will include the name of the pediatric practice where the newborn has a scheduled visit (required by Baystate prior to infant discharge) and follow-up measures outlined.

### Measures

#### Primary outcome

The MHQP website uses a star system for rating practices on each of nine measures of pediatric clinical quality with a maximum of four stars. Similarly, the site uses a star system for rating practices on each of seven patient experience measures. A mean quality score and a mean patient experience score will be created for each practice within a 25-mile radius of the hospital that has data reported on the MHQP website for at least three relevant measures. The primary outcomes will be a) the mean MHQP clinical quality and b) mean patient experience scores of the pediatric practices selected by the participants in the navigator intervention compared to the control. All measures are summarized in Table 3.

**Table 3:** Summary of outcome and other measures

	**When completed**	
	**Baseline**	**Post-intervention**
**Primary outcome**		
Clinical quality and patient experience scores of pediatric practice selected for child’s newborn visit		X
**Secondary outcome**		
Importance of factors in selecting a pediatric practice	X	X
PAM/P-PAM	X	X
Challenges encountered using website data		X
**Other factors assessed**		
Demographics (for example, age, race/ethnicity, marital status)	X	
Awareness of health care quality measures	X	X
Use of publicly reported quality data	X	X
NVS - health literacy	X	
NVS - numeracy	X	

*PAM* Patient Activation Measure, *P-PAM* Parental Patient Activation Measure, *NVS* Newest Vital Sign.

#### Secondary outcomes

**Patient activation measures** The Patient Activation Measure (PAM) [30] and Parental Patient Activation Measure (P-PAM) [31] are valid, reliable, uni-dimensional, probabilistic Guttman-like scales that reflect a developmental model of activation. The measures can be used to assess changes in individuals’ levels of activation for self-management of health care [30,31]. Prior studies have shown individual and parental activation can differ for the individual, with parental activation being higher than individual [31]. Interventions with decision aids and to improve access to health care have each improved activation measure scores [32,33].

**Importance of factors in selecting a pediatric practice** As part of the study survey, participants will be asked to rate the importance of several factors in selecting a pediatric practice. Factors will be rated on a five-point Likert scale and will include recommendations of friends or family, advice from a physician, an existing relationship with a provider, insurance, and proximity to home.

#### Other measures

**Demographics** Demographic data collected will include age, race/ethnicity, country of birth, parity, household income, marital status, number of adults in the household, number of children in the household, insurance status, primary language spoken at home, and name of current pediatric practice if the participant has children.

**Use of publicly reported data** As part of the study survey, we will determine participants’ familiarity with health care quality measures, variation in quality of care, awareness of publicly reported health care quality data, and their personal use of publicly reported health care quality data outside of the study.

**Health literacy and numeracy** The Newest Vital Sign (NVS) is a screening tool that assesses general literacy and numeracy skills as applied to health information. Individuals examine information on a nutrition label and answer six questions about how they would interpret and act on that information. The NVS takes approximately 3 minutes to complete.

**Challenges encountered using website data to guide provider choice** At the post-delivery interview, women will be asked what difficulties, if any, they encountered acting upon the information they accessed either with the navigator or on their own using the information pamphlet. Anticipated barriers include limitations imposed by the participant’s insurance carrier and transportation difficulties.

### Analysis

#### Intervention efficacy

Descriptive statistics will be used to characterize the study population and evaluate the distributions of outcomes, predictors and covariates. We will use *t*-tests and Wilcoxon rank-sum tests to compare mean quality and patient experience scores of the pediatric practices selected between women randomized to the navigator intervention and the information pamphlet control. This analysis will be both overall and within strata defined by parity (0, 1+). Our primary analysis will be intent-to-treat, based upon randomization, whether or not the intervention sessions occurred. Patients who choose practices that do not appear on the MHQP website will not be included in the primary outcome analysis but will be included in secondary outcome analyses. Secondary analysis will adjust for compliance with the intervention. We will also evaluate analysis of variance models for the mean quality and patient experience scores, adjusting for parity and any baseline patient characteristics that were not balanced between the intervention groups. An interaction effect for intervention with parity will be included to test for difference in the intervention efficacy between women selecting a pediatrician for the first time versus those with children. Confounding by covariates will be assessed. In addition we will explore the impact of clustering of women within neighborhoods defined by residential zip code, since location of practice relative to home may have a large impact on choice of practice. Final models will provide an estimate of the patient navigator effect on the quality and patient experience ratings of selected pediatric practice, both overall and by parity, adjusting for important maternal characteristics. If practice scores on the website should change during the course of the study, the scores present during the prenatal period will be used in the analysis.

#### Intervention efficacy among subgroups

We will use *t*-tests and Wilcoxon rank-sum tests to compare mean quality and patient experience scores of pediatric practices between intervention and control arms among subgroups of women defined by age, race/ethnicity, literacy, and other baseline characteristics, both overall and stratified by parity. We will then use analysis of variance models to evaluate quality and experience scores as functions of intervention status and patient baseline characteristics. Effect modification will be evaluated by means of interaction of the intervention with selected patient baseline characteristics. Results of these models will give insight into characteristics of those mothers for whom the patient navigator intervention proved more valuable.

#### Importance of factors in choosing a pediatrician

We will evaluate the distribution of responses to the five-point scale (‘Did not matter at all’ to ‘Mattered a lot’) for each factor in choosing a pediatrician at baseline and follow up. We will compute a within-subject change in importance for each factor, using t-tests and sign tests to determine whether there is a change from baseline and follow up. Initial analyses will be stratified by parity.

#### Impact of intervention on patient and parent activation to self-manage health care

We will evaluate differences in patient and parental activation change between control and intervention by means of *t*-tests or Wilcoxon rank-sum tests. Analysis of variance models will be used to evaluate change in activation, adjusting for baseline activation levels as well as other patient. Initial models will include an interaction effect for intervention with parity. Results from these models will provide information on the impact of the intervention on changing patient and parental health attitudes, whether or not there was an impact on the quality scores of practices selected.

### Power/sample size

Power was evaluated to detect a difference between intervention groups in mean quality and patient experience ratings of pediatric practices, within strata defined by parity (0, 1+). In an 18-month period, we anticipate 2,250 pregnant women will be seen at the Wesson Women’s OB and Midwifery Clinic. Based upon prior experience with research recruitment in these clinics, we expect to be able to approach about two thirds, or approximately 1,500 women. Of these we anticipate 1,200 will meet age and language eligibility requirements and that 730 will agree to participate, with 650 completing baseline and post-delivery data collection.

Expecting approximately 40% nulliparous women [28], we will need to have at least 130 women in each study arm within strata defined by parity. Quality data were identified on the MHQP website for 18 practices within a 25-mile radius of Springfield, with a mean (SD) quality score of 3.1 (0.5) on nine pediatric measures. Mean (SD) patient experience scores were similar at 3.0 (0.6). Using a significance level of 0.01, we will have power greater than 80% to detect a mean difference of 0.2 units in quality scores between control and intervention, within each stratum of parity. Power is greater than 80% to detect a 0.25 difference in mean patient experience ratings. The magnitude of the differences (0.2, 0.25) would indicate a meaningful difference in mean quality or patient experience rating of practices selected, yet this difference is large enough that we expect it could be detected.

## Discussion

In this paper, we describe our rationale and methods for an RCT to test the efficacy of a patient navigator intervention for increasing the impact of publicly reported quality of care data on the choice of a pediatric care provider. The potential impact of this study is great. With more than 73 million children under the age of 18 residing in the US [34], the quality of ambulatory pediatric care can have an enormous impact on population health.

The impact of publicly reported quality data on patients’ choice has been disappointing, due in part to challenges in communicating the information to patients in a meaningful way [14]. Our study takes a novel approach to making the science of public reporting available and understandable to a population known to be at greatest risk for limited capacity to use these data. Two elements of this study design enhance the potential for success: use of patient navigators and engagement of participants while they are receiving prenatal care. Although patient navigators have been used to assist patients in obtaining preventive care and treatment for complex medical conditions [22-24], we are unaware of studies in which they have been used to enhance informed decision-making about choice of providers for a low income population. Navigators have demonstrated the greatest effectiveness among populations with lower socioeconomic status and low health literacy [25-27]; thus, targeting a population with these characteristics may offer the greatest potential for effectiveness of the intervention. Pregnancy is also a time when women may be more engaged in their health and healthcare than at other times [35]. This motivation, coupled with sufficient lead time between knowing they need to select a pediatrician and selecting one, makes this an opportune time to test navigator efficacy.

If successful, this model has important ramifications. Team approaches to care are increasingly used in the ambulatory setting [36]. Although it is unlikely that a single practice can sponsor a dedicated patient navigator, it seems reasonable that case managers and ancillary staff can be trained to provide information about care quality to patients as part of a global effort to engage patients in health care decision-making. Should this efficacy trial favor use of navigators, this may have important implications for design and scope of responsibilities for care team members.

This study has limitations. First, women who already have children may be less likely to change pediatricians, even if quality scores for the current pediatrician are low. We have attempted to account for this by stratifying randomization to multiparous and nulliparous women and by asking mothers to rate the level of importance of a range of factors that may impact choice of pediatrician. Second, because the website is only available in English, we are not enrolling patients who are not comfortable with the English language. Few organizations have the resources to make websites available in multiple languages, but this continues to limit access to these data. Third, there is debate about how meaningful these types of quality measures are, with questions about the quality of the data used to develop them and the paucity of data linking them to improved patient outcomes. However, the MHQP website has been recognized for the soundness of the MHQP methods of compiling the performance scores, and there is growing use of these measures for assessment of care quality. Fourth, although we do not include a cost analysis for this intervention, it is possible that it may be prohibitively expensive to undertake outside of the study setting. If the intervention is successful, we plan to explore mechanisms of delivering the intervention in an affordable manner, such as using existing staff in obstetricians’ offices. Additionally, while the study was designed with power to detect differences within strata defined by parity, we may be underpowered to detect differences in secondary outcomes, or within subgroups of patients defined by literacy or ethnicity. It may be possible that as websites improve and become more usable for patients with limited health literacy, the need for a navigator may become less important.

In this paper, we have given a brief description of the rationale for a patient navigator intervention to increase access and understanding of publicly reported pediatric quality data for low-income pregnant women. This study will help determine whether removing barriers to use of quality-of-care data impacts women’s choice of pediatrician. If navigators are found to be effective in this study, patient navigator effectiveness in settings with other vulnerable populations may be warranted.

## Trial status

Piloting of study materials, intervention, and navigator training are complete and we are actively enrolling patients.

## Abbreviations

BMC: Baystate Medical Center; MHQP: Massachusetts Health Quality Partners; NVS: Newest Vital Sign; PAM: Patient Activation Measure; PI: Principal investigator; P-PAM: Parental Patient Activation Measure; RCT: Randomized controlled trial; TUSM: Tufts University School of Medicine.

## Competing interests

The authors declare that they have no competing interests.

## Authors’ contributions

SG, PL, PP, KM, TL, and KW contributed to the study design. PP was primarily responsible for the analytic plan. SG wrote the manuscript with contributions from PL, PP, TC, KM, TL, and KW. All authors read and approved the final manuscript.

## References

[B1] Hamilton BE Martin JA Ventura SJ National vital statistics reports. Births: preliminary data for 2009 The Centers for Disease Control 2010 59 3 http://www.cdc.gov/nchs/data/nvsr/nvsr59/nvsr59_03.pdf 25073731

[B2] Child trends data bank Well-Child Visits 2010 http://www.childtrendsdatabank.org/?q=node/85

[B3] Brown B Weitzman M Bzostek S Kavanaugh M Aufseeser D Bagley S Berry D Auinger P Early child development in social context: a chartbook The Commonwealth Fund http://www.commonwealthfund.org/usr_doc/ChildDevChartbk.pdf

[B4] National institutes of health Medline plus Well-child Visits 2011 http://www.nlm.nih.gov/medlineplus/ency/article/001928.htm

[B5] Chung PJ Lee TC Morrison MA Schuster MA Preventive care for children in the United States: quality and barriers Annu Rev Public Health 2006 27 491 551 10.1146/annurev.publhealth.27.021405.102155 16533127

[B6] Hakim RB Ronsaville DS Effect of compliance with health supervision guidelines among U.S. infants on emergency department visits Arch Pediatr Adolesc Med 2002 156 1015 1020 10.1001/archpedi.156.10.1015 12361448

[B7] Rosenberg SA Zhang D Robinson CC Prevalence of developmental delays and participation in early intervention services for young children Pediatrics 2008 121 e1503 e1509 10.1542/peds.2007-1680 18504295

[B8] Sice L Developmental screening in primary care: the effectiveness of current practice and recommendations for improvement The Commonwealth Fund 2007 http://www.commonwealthfund.org/usr_doc/1082_sices_developmental_screening_primary_care.pdf?section=4039

[B9] Rising to the challenge: results from a scorecard on local health system performance Commonwealth Fund 2012 http://www.commonwealthfund.org/Publications/Fund-Reports/2012/Mar/Local-Scorecard.aspx

[B10] National committee for quality assurance report: health care quality improves but varies across different regions of the country http://www.ncqa.org/tabid/847/Default.aspx

[B11] Collins MM O’Sullivan T Harkins V Perry IJ Quality of life and quality of care in patients with diabetes experiencing different models of care Diabetes Care 2009 32 4 603 605 10.2337/dc08-1169 19171727 PMC2660479

[B12] Henry J Kaiser Family Foundation 2008 Update on Consumers’ views of patient safety and quality information 2008 2400 Sand Hill Road, Menlo Park, CA 94025 Publication #7819

[B13] Berwick DM James B Coye MJ Connections between quality measurement and improvement Med Care 2003 41 Suppl 1 130 138 10.1097/00005650-200301001-00004 12544814

[B14] Ketelaar NA Faber MJ Flottorp S Rygh LH Deane KH Eccles MP Public release of performance data in changing the behaviour of healthcare consumers, professionals or organisations Cochrane Database Syst Rev 2011 11 CD004538 10.1002/14651858.CD004538.pub2 PMC4204393 22071813

[B15] Hibbard J Sofaer S Agency for Healthcare Research and Quality Best practices in public reporting No. 3: How to maximize public awareness and Use of comparative quality reports through effective promotion and dissemination strategies AHRQ publication No. 10-0082-EF 2010 Rockville, MD: Agency for Healthcare Research and Quality http://www.ahrq.gov/legacy/qual/pubrptguide3.htm

[B16] Hibbard J Sofaer S Agency for Healthcare Research and Quality Best practices in public reporting No. 1: How to effectively present health care performance data to consumers. AHRQ publication No. 10-0082-EF 2010 Rockville, MD: Agency for Healthcare Research and Quality http://www.ahrq.gov/legacy/qual/pubrptguide1.htm

[B17] Hibbard JH Peters E Dixon A Tusler M Consumer competencies and the use of comparative quality information: it isn’t just about literacy Med Care Res Rev 2007 64 379 394 10.1177/1077558707301630 17684108

[B18] Hibbard JH Jewett JJ Will quality report cards help consumers? Health Aff (Millwood) 1997 16 218 228 9141339 10.1377/hlthaff.16.3.218

[B19] Hibbard JH Greene J Daniel D What is quality anyway? performance reports that clearly communicate to consumers the meaning of quality of care Med Care Res Rev 2010 67 275 293 10.1177/1077558709356300 20093399

[B20] Bardach NS Hibbard HJ Dudley RA Agency for Healthcare Research and Quality Users of public reports of hospital quality: who, what, why, and how? an aggregate analysis of 16 online public reporting Web sites and users’ and experts’ suggestions for improvement 2011 Rockville, MD: Agency for Healthcare Research and Quality AHRQ Publication No. 12-0016-EF

[B21] Massachusetts health quality partners, quality insightshttp://www.mhqp.org/default.asp?nav=010000

[B22] Phillips CE Rothstein JD Beaver K Sherman BJ Freund KM Battaglia TA Patient navigation to increase mammography screening among inner city women J Gen Intern Med 2011 26 123 129 10.1007/s11606-010-1527-2 20931294 PMC3019333

[B23] Battaglia TA Roloff K Posner MA Freund KM Improving follow-up to abnormal breast cancer screening in an urban population. A patient navigation intervention Cancer 2007 109 Suppl 2 359 367 17123275 10.1002/cncr.22354

[B24] Hunnibell LS Rose MG Connery DM Gren CE Hampel JM Rosa M Vogel DC Using nurse navigation to improve timeliness of lung cancer care at a veterans’ hospital Clin J Oncol Nurs 2012 16 29 36 10.1188/12.CJON.29-36 22297004

[B25] Lopez L Grant RW Closing the gap: eliminating health care disparities among Latinos with diabetes using health information technology tools and patient navigators J Diabetes Sci Technol 2012 6 169 176 22401336 10.1177/193229681200600121 PMC3320835

[B26] Hendren S Chin N Fisher S Winters P Griggs J Mohile S Fiscella K Patients’ barriers to receipt of cancer care, and factors associated with needing more assistance from a patient navigator J Natl Med Assoc 2011 103 701 710 22046847 10.1016/s0027-9684(15)30409-0 PMC3713073

[B27] Natale-Pereira A Enard KR Nevarez L Jones LA The role of patient navigators in eliminating health disparities Cancer 2011 117 Suppl 15 3543 3552 21780089 10.1002/cncr.26264 PMC4121958

[B28] Chasan-Taber L Silveira M Marcus BH Braun B Stanek E Markenson G Feasibility and efficacy of a physical activity intervention among pregnant women: the behaviors affecting baby and you (B.A.B.Y.) study J Phys Act Health 2011 8 Suppl 2 S228 S238 28829712 10.1123/jpah.8.s2.s228

[B29] Yudkowsky BK Tang SS Siston AM American Academy of Pediatrics Division of Health Policy Research. Pediatrician Participation in Medicaid/SCHIP. Survey of the Fellows of the American Academy of Pediatrics 2000 Health http://www2.aap.org/research/med-schip/dpc/ma.pdf

[B30] Hibbard JH Stockard J Mahoney ER Tusler M Development of the patient activation measure (PAM): conceptualizing and measuring activation in patients and consumers Health Serv Res 2004 39 1005 1026 10.1111/j.1475-6773.2004.00269.x 15230939 PMC1361049

[B31] Pennarola BW Rodday AM Mayer DK Ratichek SJ Davies SM Syrjala KL Patel S Bingen K Kupst MJ Schwartz L Guinan EC Hibbard JH Parsons SK Factors associated with parental activation in pediatric hematopoietic stem cell transplant Med Care Res Rev 2011 69 194 214 22203645 10.1177/1077558711431460 PMC4160822

[B32] McDonald EM Frattaroli S Edsall Kromm E Ma X Pike M Holtgrave D Improvements in health behaviors and health status among newly insured members of an innovative health access plan J Community Health 2013 38 301 309 10.1007/s10900-012-9615-3 23014801

[B33] Den D Lu WH Weintraub MR Maranda MJ Elshafey S Gold MR The impact of different modalities for activating patients in a community health center setting Patient Educ Couns 2012 89 178 183 10.1016/j.pec.2012.04.012 22683294

[B34] US Department of Commerce: United States Census Bureau QuickFactshttp://quickfacts.census.gov/qfd/states/00000.html

[B35] Heppner WL Ji L Reitzel LR Castro Y Correa-Fernandez V Vidrine JI Li Y Dolan-Mullen P Velasquez MM Cinciripini PM Cofta-Woerpel L Greisinger A Wetter D The role of prepartum motivation in the maintenance of postpartum smoking abstinence Health Psychol 2011 30 736 745 21859215 10.1037/a0025132 PMC3221324

[B36] Blumenthal DM Song Z Jena AB Ferris TG Guidance for structuring team-based incentives in healthcare Am J Manag Care 2013 19 e64 e70 23448116 PMC3984877

